# Interface Analysis between Inconel 625 and Cobalt-Chromium Alloy Fabricated by Powder Bed Fusion Using Pulsed Wave Laser

**DOI:** 10.3390/ma16196456

**Published:** 2023-09-28

**Authors:** Liming Yao, Aditya Ramesh, Zongheng Fan, Zhongmin Xiao, Guanhai Li, Quihui Zhuang, Jing Qiao

**Affiliations:** 1 School of Mechanical and Aerospace Engineering, Nanyang Technological University, 50 Nanyang Avenue, Singapore 639798, Singapore; 2 State Key Laboratory of Infrared Physics, Shanghai Institute of Technical Physics, Chinese Academy of Sciences, Shanghai 200083, China; ghli0120@mail.sitp.ac.cn; 3 School of Mechanical Engineering, Chongqing University of Technology, Chongqing 400054, China; 4 School of Materials Science and Engineering, Harbin Institute of Technology, Harbin 150080, China; jingqiao@hit.edu.cn; 5 Zhengzhou Research Institute of Harbin Institute of Technology, Zhengzhou 450002, China

**Keywords:** additive manufacturing, dissimilar alloy, interface strength, Pulsed Wave Laser

## Abstract

A few components used in the aerospace and petrochemical industries serve in corrosive environments at high temperatures. Corrosion-resistant metals or unique processes, such as coating and fusion welding, are required to improve the performance of the parts. We have used laser powder bed fusion (LPBF) technology to deposit a 5 mm thick corrosion-resistant CoCrMo layer on a high-strength IN625 substrate to improve the corrosion resistance of the core parts of a valve. This study found that when the laser volumetric energy density (*E*_V_) ≤ 20, the tensile strength increases linearly with the increase in *E*_V_, and the slope of the curve is approximately 85°. The larger the slope, the greater the impact of *E*_V_ on the intensity. When *E*_V_ > 20, the sample strength reaches the maximum tensile strength. When the *E*_V_ increases from 0 to 20, the fracture position of the sample shifts from CoCrMo to IN625. When *E*_V_ ≤ 38, the strain increases linearly with the increase in *E*_V_, and the slope of the curve is approximately 67.5°. The sample strain rate reaches the maximum when *E*_V_ > 38. Therefore, for an optimal sample strength and strain, *E*_V_ should be greater than 38. This study provides theoretical and technical support for the manufacturing of corrosion-resistant dissimilar metal parts using LPBF technology.

## 1. Introduction

**Figure 1:**
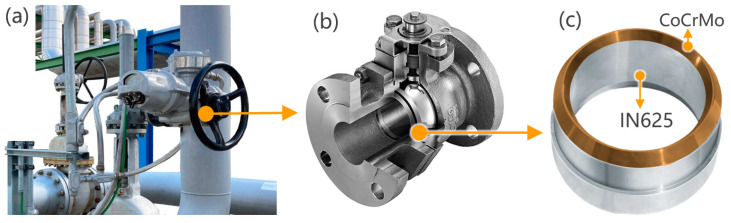
(**a**) Pipeline systems in the chemical industry; (**b**) ball valve; (**c**) valve seat ring.

Most of the components in the aerospace, nuclear and petrochemical industries work in harsh and challenging environments, making them extremely vulnerable to corrosion and thermal fatigue [1,2,3]. For example, the chemical plant maintenance team at a large global petrochemical company noticed a requirement for frequent repairs and replacement of valve components, since the plant was operating with acidic fluids with solid-liquid two-phase flow, see Figure 1. This resulted in excessive downtime [4,5,6]. The common methods to enhance corrosion resistance include cold/hot coating, fusion welding, etc. However, for long-serving components such as valves, the above-mentioned dissimilar alloy joining technologies are usually ineffective. This is attributed to the thin and erosion-prone nature of corrosion-resistant alloy coatings, along with weak interfaces between the alloys. LPBF is a feasible alternative to firmly combine the two metals [7,8,9]. To enhance the lifespan of the existing valves, we employed LPBF technology to deposit a 5 mm thick CoCrMo layer onto the base of an IN625 (Inconel 625) valve core. This will significantly enhance their durability and performance in corrosive environments [10,11,12]. Therefore, this technology represents a significant advancement in safeguarding the integrity of petrochemical processes and reducing the economic risks associated with corrosion-related failures [13,14,15].

LPBF enables the fabrication of multi-metal components by selectively depositing different metal powders in precise patterns [16,17]. Various metal combinations can be explored to create synergistic effects that enhance corrosion resistance [18]. For example, stainless steel combined with tungsten-based alloys can provide excellent corrosion resistance in marine and chemical environments [19]. It can also be combined with chromium to improve its corrosion resistance and passivation capacity [20]. Titanium integrated with aluminium can create components with enhanced corrosion resistance for biomedical or electronic applications [21,22]. In the study conducted by Fu et al., SUS301L-MT cold rolled stainless steel served as the base material. Laser-arc hybrid welding (LAHW) was used for the joining process. They observed that in base metal and heat-affected zones (HAZs), hydrogen-induced cracks initiated at the α′/γ interface and propagated along the austenite grain boundaries. The cracks tend to start in regions with a higher hydrogen concentration and strain, followed by microcrack formation at the crack tip due to hydrogen-enhanced local plastic deformation or hydrogen-enhanced decohesion mechanisms, ultimately leading to failure due to hydrogen embrittlement [23,24]. At present, most commercial LPBF systems use continuous wave (CW) lasers as the main heating source. A limited number of industrial systems prefer to use a pulsed wave (PW) laser as the heating source [25,26,27].Based on the specific application, there are significant advantages to both of these types of lasers. When the duty cycle of the PW laser is less than 1.0, a small melt pool with a large temperature gradient is created. Conversely, a PW laser with a duty cycle of 1.0 is essentially equivalent to a CW laser, thus resulting in a larger melt pool and slower cooling rates. The variation in heat input between PW and CW lasers leads to the differences in microstructure and material properties of metals [15,28,29,30]. The precise control over laser energy and the ability to create short, intense pulses help minimize the HAZ in LPBF, thus reducing the risk of thermal distortion and stress. It also contributes to smoother surface finishes on printed parts due to their ability to produce fine melt tracks. Process parameters, including pulse duration, pulse energy, and scanning speed, must be carefully optimized for specific materials and part geometries to achieve the desired results [31,32,33].

Corrosion-resistant valves are vital assets within the petrochemical industry, serving as critical components in the safe and efficient operation of various processes. They play a pivotal role in safeguarding the integrity of petrochemical infrastructures. Corrosion-related valve failures can lead to unplanned shutdowns and production interruptions, resulting in substantial financial losses. Corrosion-resistant valves reduce the risk of such occurrences, ensuring continuous and efficient production processes. The innovative application of LPBF with a PW laser to selectively clad CoCrMo onto IN625 valves creates highly customized corrosion-resistant coatings [34,35,36]. CoCrMo is chosen for its exceptional resistance to corrosion, enhancing the longevity and reliability of IN625 valves, which are renowned for their high-temperature and corrosion-resistant properties. However, the mechanical properties of IN625/CoCrMo interfaces fabricated with the help of commercial PW lasers have not been studied in the existing literature. The effect of PW lasers on multi-metal LPBF has also not been studied in extensive detail [37,38]. Moreover, there are not many studies on the mechanical and metallurgical characteristics of IN625/CoCrMo interfaces fabricated by Selective Laser Melting (SLM). The primary factor affecting the strength of the IN625/CoCrMo interface is the input laser energy density. Insufficient melting of the CoCrMo powder due to a lower laser energy density may lead to bonding failure. High laser energy density may cause the formation of pores along the IN625/CoCrMo interface, thereby reducing product quality and production efficiency.

In this paper, we studied the relationship between the volumetric energy density and tensile strength of an IN625/CoCrMo interface fabricated via LPBF using PW lasers. We have highlighted the effect of the change in process parameters (viz. laser power, point distance, scan velocity and exposure time) on the tensile strength and volumetric energy density. We have also extensively analysed the relationship between the effect of remelting the first three layers of powder over the substrate and the maximum tensile strength. Finally, we aim to find the optimum volumetric energy density to fabricate a corrosion-resistant valve with high strength.

## 2. Materials and Methods

### 2.1. LPBF Process Parameters

**Figure 2:**
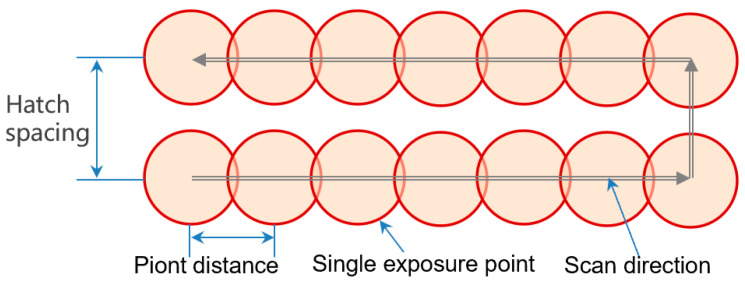
Working principle and key parameters of pulsed wave laser.

We used a Renishaw AM400 LPBF device (Renishaw, Tokyo, Japan) to print CoCrMo blocks on the IN625 substrate. The particle size range of CoCrMo powder is 10–45 µm. The AM400 uses a fibre laser with a wavelength of 1070 nm and a laser diameter of 70 μm, operating in a power-modulated PW mode. In PW mode, AM400 can control laser power, point distance, exposure time and hatch spacing (Figure 2). Table 1 lists the 27 process parameter combinations used in this experiment. Scan velocity *V* is the ratio of point distance (*D*_p_) to exposure time (*E*_t_) and ranges from 0.36 to 2.00 m/s. The laser scan direction was rotated by 90° after each layer of powder was printed, and no boundary scan was applied. In this study, the powder layer thickness (*T*) is 60 µm, and the hatch distance ($H_{s}$) is 80 µm. In comparison with the CW laser, the energy input of the PW laser is intermittent. The volumetric energy density is an important parameter to measure the laser energy input. For the *E*_V_ of a CW laser emitter, Equation (1) is to be referred to [32]. The high-quality part printing parameters for CoCrMo powder are *P* = 200, *D*_p_ = 70 μm and *E*_t_ = 80 μs. Using the values mentioned above, regular parameters are selected within a certain range for printing and testing. The objective is to identify a high-strength parameter combination for the interface between CoCrMo and IN625. For pulsed laser transmitters, Equation (1) needs to be modified to Equation (2) to quantify the parameter combinations shown in Table 1 [39].

(1) $$E_{V}=P/\left(T\times H_{s}\times V\right)$$

(2) $$E_{V}=P\times \delta /\left(T\times H_{s}\times V\right)$$

In the above equations, *P* denotes the laser power, $H_{s}$ is the hatch spacing, *V* is the scan velocity, $D_{p}$ is the point distance, and $E_{t}$ refers to the exposure time. Equation (2) adds the duty cycle term *δ*, ranging from 0.0 and 1.0, which serves as a multiplier to account for the PW exposure parameter. According to the study by Brown et al., the exposure times of 50 μs, 80 μs, and 110 μs correspond to duty cycles of 0.54, 0.75, and 0.90, respectively [13]. The $E_{V}$ was calculated by Equation (2) and is listed in Table 1.

When printing CoCrMo onto IN625 substrate, the differences in thermophysical properties and composition between the two metal alloys may weaken the areas near the interface. We executed the process parameters listed in Table 1 to examine this concept through the following two approaches. The initial approach entails printing the powder layer by layer without laser remelting, while the second approach involves remelting the initial three layers of powder once, with no remelting operation for the subsequent powder layers. 

### 2.2. Tensile Test

**Figure 3:**
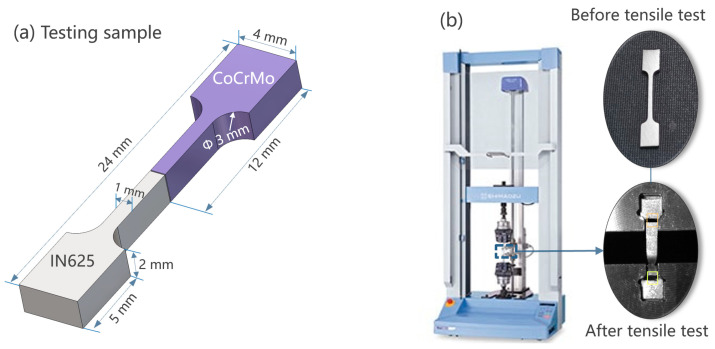
Schematic of (**a**) sample dimensions and (**b**) equipment for tensile testing.

To enhance the surface quality and eliminate visible surface cracks, all samples underwent initial polishing using 220 and 500 SiC grit papers before the commencement of the tensile tests. The ultimate tensile strength of the polished tensile samples was analysed using a Shimadzu Autograph AG-X Plus 10Kn (Shimadzu, Kyoto, Japan) (Figure 3). Fixtures were securely attached to the upper and lower arms of the tensile machine holders. The gauge length of the tensile sample was set to 1 cm. By employing a non-contact digital video extensometer called TRViewX (Shimadzu, Kyoto, Japan) along with specialized software, optimal lighting conditions were maintained for various specimen types. With an exceptional precision of 1.5 µm, the extensometer could detect even the slightest changes in specimen elongation. Calibration ensured an accurate measurement of the longitudinal strain between the designated markers. Vernier callipers were utilized to determine the width and thickness of the tensile samples, and these measurements were entered into the software. Pre-loading of the sample to 10 N was conducted. Subsequently, a steadily increasing tensile force was applied until the sample reached the point of fracture. This procedure was iterated for all 54 samples, and the fracture location for each specimen was documented. Fractures could either occur towards the IN625 or CoCrMo end of the sample, or precisely at the interface. We printed two samples for the tensile testing of each set of process parameters listed in Table 1. The average of the two tests is shown in Table 1.

## 3. Results and Discussion 

**Figure 4:**
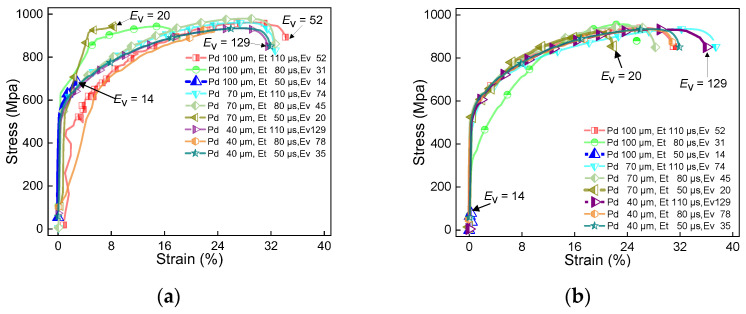
Stress–strain curve of IN625/CoCrMo sample with laser power of 250 W. (**a**) No remelting of the first three layers of powder above the substrate; (**b**) laser remelting of the first three layers of powder above the substrate.

**Figure 5:**
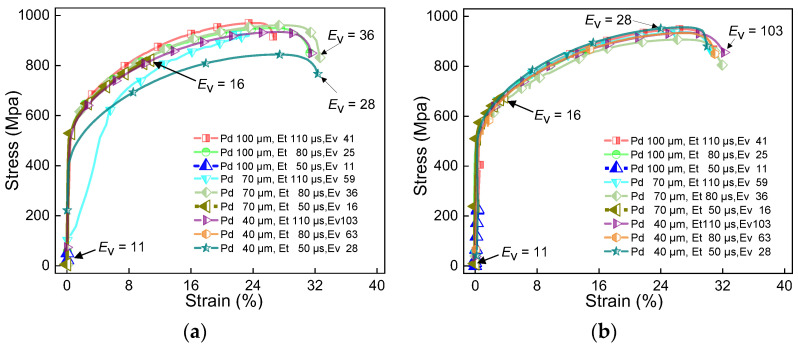
Stress–strain curve of IN625/CoCrMo sample with laser power of 200 W. (**a**) No remelting of the first three layers of powder above the substrate; (**b**) laser remelting of the first three layers of powder above the substrate.

**Figure 6:**
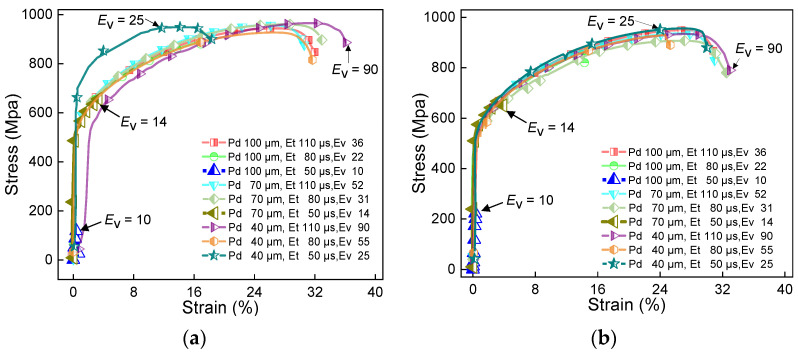
Stress–strain curve of IN625/CoCrMo sample with laser power of 175 W. (**a**) No remelting of the first three layers of powder above the substrate; (**b**) laser remelting of the first three layers of powder above the substrate.

**Figure 7:**
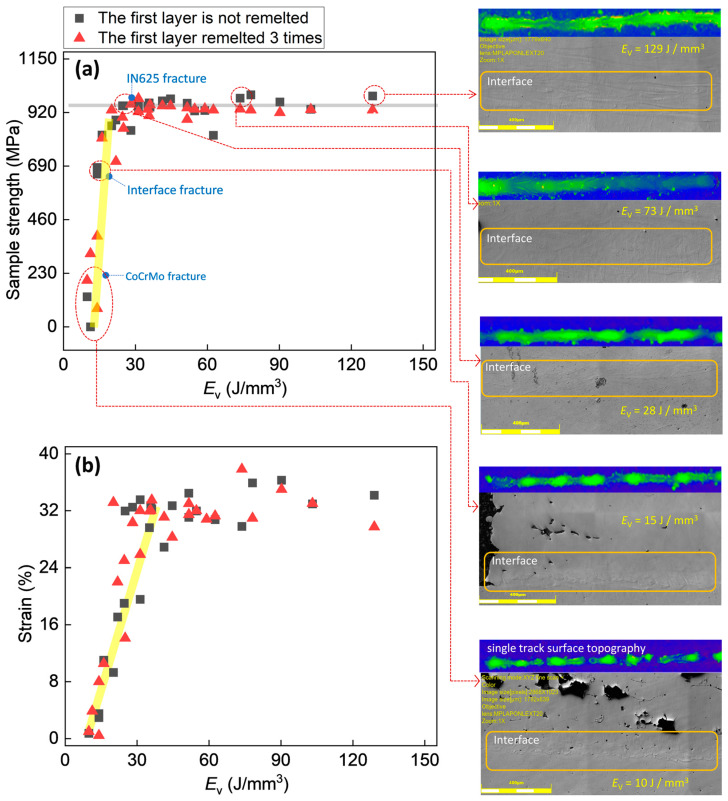
Variation of (**a**) tensile strength and (**b**) strain of IN625/CoCrMo sample with volumetric energy density *E*_V_. The right side of (**a**,**b**) represents the single-track surface topography (blue-green images) and pores (gray images) near the interface, corresponding to different *E*_V_. The light-gray thick line in (**a**) represents the limiting strength of pure IN625 (940 MPa). The light-yellow thick line in (**a**,**b**) represents the trend of the stress and strain variation.

Since each material has a specific laser absorbance and thermal properties, extensive experimental work must be carried out to obtain the processing windows for novel material systems. These processing parameters are crucial to produce parts with desired properties. Figure 4, Figure 5 and Figure 6 show the stress–strain curves of samples under different parameters. Figure 7 shows the effect of volumetric energy density on stress and strain.

Figure 4, Figure 5 and Figure 6 show the stress–strain curves under different laser powers. These figures analyse the effect of laser power on the performance of the sample. In Figure 4, Figure 5 and Figure 6, (a) represents the samples with no remelting of any layers, and (b) represents the samples whose first three layers above the substrate were remelted. This was done to analyse the effect of laser scanning strategies on the sample performance. By comparing (a) and (b) in Figure 4, Figure 5 and Figure 6, it can be observed that a particular pattern is followed by the stress–strain curves, irrespective of the remelting of the first three layers of CoCrMo powder. When *E*_V_ ≤ 20, the volumetric energy density has a significant impact on the tensile strength, ranging from 40 MPa to 940 MPa. However, the overall strain values are relatively small at this stage. From the curves, it can be inferred that a higher *E*_V_ results in a higher tensile strength. When *E*_V_ ≤ 38, it has a greater impact on strain, ranging from 10% to 30%. When *E*_V_ > 38, the stress–strain behaviour becomes more stable, and the maximum tensile strength is extremely close to the ultimate tensile strength of pure IN625 (i.e., 940 MPa). The detailed strength and strain results can be obtained from Table 1.

If we compare the curves in Figure 7a,b, we can observe that the stress–strain curves after remelting are more stable, and the curves are clustered more tightly. For instance, in Figure 4a, the stress–strain curves at *E*_V_ = 20, 31, 52, and 78 deviate from the clustered curves. In Figure 4b, only the curve with *E*_V_ = 31 deviates from the clustered curves. Similar patterns are also observed in Figure 5 and Figure 6. Since remelting promotes the fusion of the two metals near the interface, the transition of physical properties near the interface is smoother, resulting in more concentrated stress–strain curves of the first three layers of remelted samples. When *E*_V_ ≤ 20, inadequate powder melting makes the bonding of the two metal alloys near the interface more challenging. Remelting enhances the diffusion and bonding between the two metal alloys, thus improving the interface quality. Therefore, the stress–strain curves after remelting are more tightly clustered than the ones with no remelting. However, in a few cases, we observed that the stress–strain curves primarily follow the mechanical properties of IN625 and are less influenced by the interface region.

For a more detailed understanding of the effect of *E*_V_ on the stress and strain of the samples, we used the data from Figure 4, Figure 5 and Figure 6 to create Figure 7. From Figure 7a, it can be observed that when *E*_V_ ≤ 20, the tensile strength increases linearly with the increase in *E*_V_, and the slope of the line is close to 85° (light-yellow line). When *E*_V_ > 20, the sample strength reaches the ultimate tensile strength of IN625, and subsequently it fractures. Sample analysis (laser microscopy images on the right side of Figure 7a) reveals that when *E*_V_ ≤ 15, the powder is not fully melted, resulting in fracture near the interface of the CoCrMo end. When 15 < *E*_V_ ≤ 20, fracture occurs near the interface. This is due to the increased volumetric energy density, leading to more uniform and increased powder melting, thereby enhancing the strength of CoCrMo. At this point, the strength of the sample near the interface is lower as compared to IN625 and CoCrMo. This is due to the incomplete melting and intermixing of the two metal alloys. When *E*_V_ > 20, fracture occurs towards the IN625 side, since the volumetric energy density is sufficiently high to fully melt the CoCrMo powder, thus resulting in a densely-packed-CoCrMo three-dimensional sample. In this case, the IN625 side becomes the weaker region. In Figure 7b, when *E*_V_ ≤ 38, the strain increases linearly with the increase in *E*_V_, and the slope of the line is close to 67.5° (light-yellow line). When *E*_V_ > 38, the strain reaches the ultimate tensile stress of IN625 and subsequently fractures.

Wen et al. used LPBF to fabricate compositionally graded alloy (CGA) (viz. CoCrMo/Inconel 718) similar to our research samples [40]. In their study, they introduced a novel approach for the LPBF of large-scale alloy components with continuous compositional gradients along their length. This method was demonstrated by successfully manufacturing defect-free CoCrMo-Nickel-based CGA superalloy coupons with smooth end-to-end variations in composition and microstructure. To explain the variations in tensile properties and hardness measured using high-throughput characterization techniques, they conducted detailed microscopic analysis of the various phases present in the microstructures. Their meticulous research significantly contributes to the comprehension and analysis of the results presented in this paper. Our current paper analyses the effects of scanning parameters and strategies on the strength and strain of the CoCrMo/IN625 interface. In future research, we will focus on analysing the effects of the alloying degree and microstructure on the fracture mechanism.

## 4. Conclusions

In this study, we found the ideal volumetric energy density to deposit a layer of CoCrMo on an IN625 valve via LPBF. CoCrMo powder was printed on an IN625 substrate. A total of 27 printing parameters and 2 melting modes were studied. Detailed analysis on the relation between *E*_V_ and tensile strength was presented. The changes in the stress–strain curves of multi-metal samples and the influence of volumetric energy density on stress and strain were analysed. 

The results showed that when *E*_V_ ≤ 20, the tensile strength increases linearly with the increase in *E*_V_, and the slope of the curve is approximately 85°. When *E*_V_ > 20, the sample strength reaches its maximum and is equal to the ultimate tensile strength of IN625. Further analysis revealed that when *E*_V_ ≤ 15, the excessively low volumetric energy density results in fracture that occurs on the CoCrMo side of the interface. When 15 < *E*_V_ ≤ 20, fracture occurs near the interface due to the increased volumetric energy density. At this point, the strength near the interface is lower due to the incomplete melting and intermixing of the two metallic alloys. When *E*_V_ > 20, fractures occur on the IN625 side, since the volumetric energy density is sufficiently high to fully melt the CoCrMo powder. Therefore, the IN625 side becomes the weaker region in this case. When *E*_V_ ≤ 38, the strain increases linearly with the increase in *E*_V_, and the slope of the curve is approximately 67.5°. When *E*_V_ > 38, the maximum strain is reached, and the sample subsequently fractures. The observations and conclusions summarised in this study can be effectively used to fabricate corrosion-resistant valves operating in petrochemical industries under highly acidic conditions.

## Figures and Tables

**Table 1:** LPBF process parameters and test result.

*P* (W)	*D*_p_ (μm)	*E*_t_ (μs)	*V* (m/s)	*E*_V_ (J/mm^3^)	Strength (MPa)		Strain (%)	
					No Remelting	Remelting	No Remelting	Remelting
250	100	110	0.91	52	960	940	34	31
		80	1.25	31	942	924	20	26
		50	2.00	14	684	79	3	0
	70	110	0.64	74	982	935	30	38
		80	0.88	45	979	948	33	28
		50	1.40	20	863	931	9	33
	40	110	0.36	129	991	931	34	30
		80	0.50	78	996	932	36	31
		50	0.80	35	948	934	30	32
200	100	110	0.91	41	970	948	27	31
		80	1.25	25	949	852	32	14
		50	2.00	11	0	314	32	4
	70	110	0.64	59	929	936	30	31
		80	0.88	36	961	906	33	32
		50	1.40	16	823	810	11	11
	40	110	0.36	103	934	933	33	33
		80	0.50	63	822	931	31	31
		50	0.80	28	843	956	32	30
175	100	110	0.91	36	943	953	32	34
		80	1.25	22	888	710	17	22
		50	2.00	10	129	200	1	1
	70	110	0.64	52	957	891	31	33
		80	0.88	31	960	980	34	32
		50	1.40	14	656	390	4	8
	40	110	0.36	90	965	920	36	35
		80	0.50	55	927	935	32	32
		50	0.80	25	949	900	19	25

## Data Availability

Not applicable.
